# An Ultra-Sensitive *Comamonas thiooxidans* Biosensor for the Rapid Detection of Enzymatic Polyethylene Terephthalate (PET) Degradation

**DOI:** 10.1128/aem.01603-22

**Published:** 2022-12-12

**Authors:** Robert F. Dierkes, Alan Wypych, Pablo Pérez-García, Dominik Danso, Jennifer Chow, Wolfgang R. Streit

**Affiliations:** a Department of Microbiology and Biotechnology, University of Hamburg, Hamburg, Germany; b Molecular Microbiology, Institute for General Microbiology, Kiel University, Kiel, Germany; University of Milano-Bicocca

**Keywords:** biosensor, reporter strain, terephthalic acid (TPA), PET degradation, hydrolases, PET-esterases, plastic degradation, polyethylene terephthalate (PET), comamonas

## Abstract

Polyethylene terephthalate (PET) is a prevalent synthetic polymer that is known to contaminate marine and terrestrial environments. Currently, only a limited number of PET-active microorganisms and enzymes (PETases) are known. This is in part linked to the lack of highly sensitive function-based screening assays for PET-active enzymes. Here, we report on the construction of a fluorescent biosensor based on *Comamonas thiooxidans* strain S23. *C. thiooxidans* S23 transports and metabolizes TPA, one of the main breakdown products of PET, using a specific tripartite tricarboxylate transporter (TTT) and various mono- and dioxygenases encoded in its genome in a conserved operon ranging from *tphC-tphA1.* TphR, an IclR-type transcriptional regulator is found upstream of the *tphC-tphA1* cluster where TPA induces transcription of *tphC-tphA1* up to 88-fold in exponentially growing cells. In the present study, we show that the *C. thiooxidans* S23 wild-type strain, carrying the sfGFP gene fused to the *tphC* promoter, senses TPA at concentrations as low as 10 μM. Moreover, a deletion mutant lacking the catabolic genes involved in TPA degradation *thphA2-A1 (*Δ*tphA2A3BA1*) is up to 10,000-fold more sensitive and detects TPA concentrations in the nanomolar range. This is, to our knowledge, the most sensitive reporter strain for TPA and we demonstrate that it can be used for the detection of enzymatic PET breakdown products.

**IMPORTANCE** Plastics and microplastics accumulate in all ecological niches. The construction of more sensitive biosensors allows to monitor and screen potential PET degradation in natural environments and industrial samples. These strains will also be a valuable tool for functional screenings of novel PETase candidates and variants or monitoring of PET recycling processes using biocatalysts. Thereby they help us to enrich the known biodiversity and efficiency of PET degrading organisms and enzymes and understand their contribution to environmental plastic degradation.

## INTRODUCTION

The global use of synthetic and fossil fuel-derived polymers for more than 80 years together with a lack of multinational concepts for re- and upcycling and circular use have led to an unprecedented and mostly irreversible accumulation of plastic of various sizes and blends in almost all ecological niches (1–5).

A promising approach to reduction of plastic wastes in- and outside the recycling chain are enzymes that are capable of breaking down the polymeric structure of plastics, facilitating their further degradation or even yielding raw-materials for bioplastics and other value-added products (6–9). However, today, we have only relatively few enzymes available acting on some of the human-made commodity polymers. A total of 145 enzymes are currently listed in the PAZy database acting on synthetic polymers, including 43 different esterases, cutinases, and lipases hydrolyzing the low crystalline and amorphous polymer polyethylene terephthalate (PET) (10). The majority of the PET active enzymes have been identified using sequence-based homology-and hidden Markov-model (HMM) searches. This resulted in a rather limited biodiversity with active PETases derived from only four bacterial and two fungal phyla. In order to uncover completely novel active PETases derived from a broader diversity, there is the need to establish highly sensitive and function-based assays for rapid screening of enzyme candidates and metagenome libraries.

**FIG 1:**
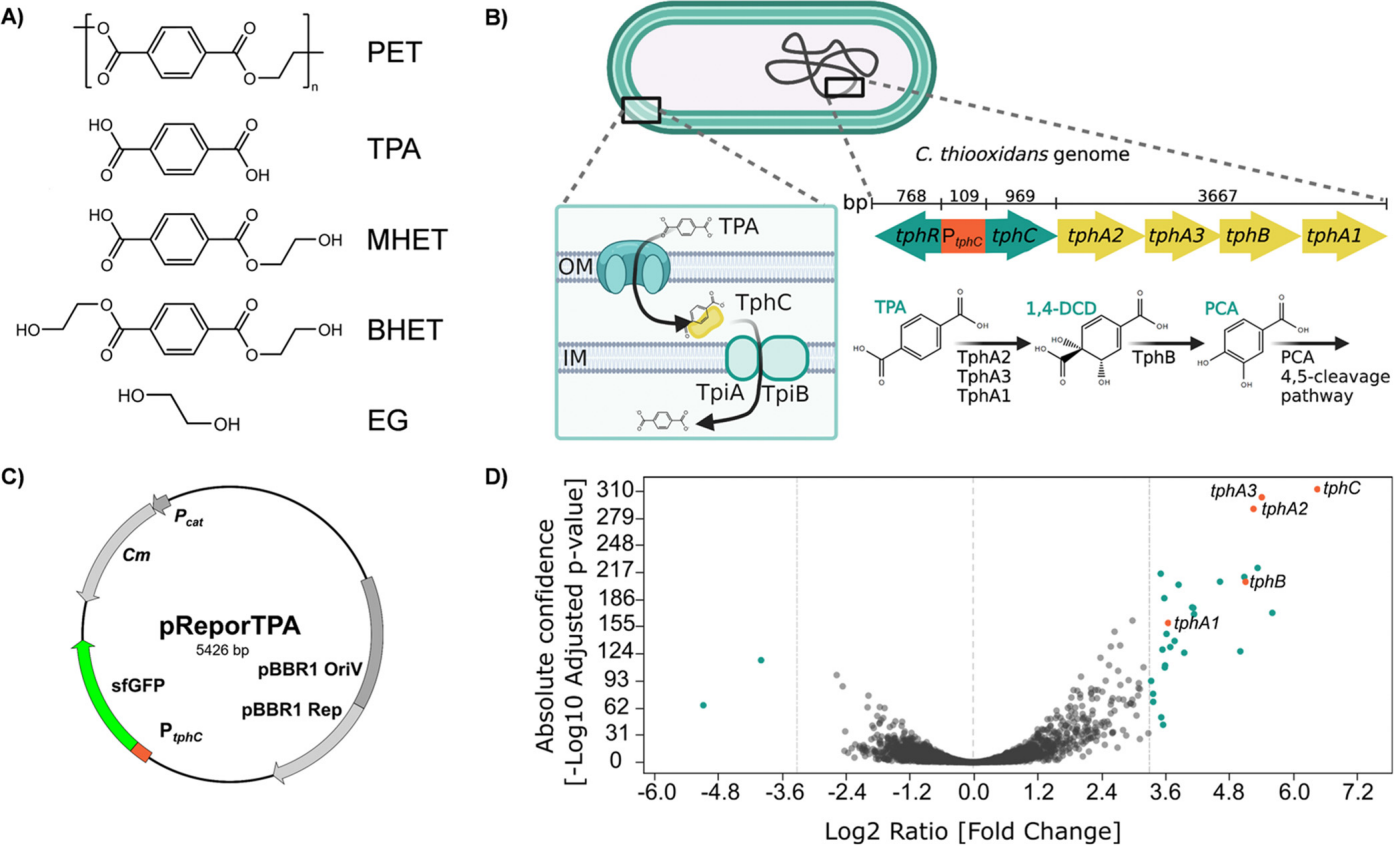
Polyethylene terephthalate (PET) with its degradation products and terephthalic acid (TPA) reporter strain construction using *C. thiooxidans* S23. (A) Enzymatic degradation products of PET: Terephthalic acid, TPA; mono(2-hydroxyethyl)terephthalate, MHET; bis(2-hydroxyethyl)terephthalate, BHET; ethylene glycol, EG. (B) Physical map of the genomic region coding for the TPA uptake and catabolic genes in *C. thiooxidans* S23. Uptake is presumably facilitated via the transporter subunits TpiA and TpiB together with the substrate binding protein TphC (26, 43). OM, outer membrane; IM, inner membrane. Degradation of TPA is regulated by TphR binding to the intergenic promoter region P*_tphC_*. Intermediates of TPA degradation are 1,2-dihydroxy-3,5-cyclohexadiene-1,4-dicarboxylate (1,4-DCD) and protocatechuate PCA. Numbers indicate the ORF and promoter length and are derived from NCBI GenBank under accession number NZ_LIOM01000016.1 (nt 48,125 – 54,351). Depiction of TPA degradation is adapted from (25.) (C) Physical map of the plasmid pReporTPA (pBBR1MCS-P*_tphC_*::sfGFP). (D) Volcano plot showing the differential expression analysis of *C. thiooxidans* grown on minimal medium containing terephthalic acid compared to growth on succinate as sole carbon source. Colored dots on the right side of the plot represent genes with a log_2_ ratio >3.32 (>~10-fold increased expression). Genes of the *tph* cluster under regulation of P*_tphC_* are indicated in orange with the respective gene names.

Currently, there are already few functional methods known to assess microbial PET degradation. These methods mainly rely on either weight loss, pH change through hydrolysis and/or on the absorption of UV-light through the aromatic constituents of the polymer. Assaying the enzymatic release of the monomeric building blocks bis(2-hydroxyethyl)terephthalate (BHET), mono(2-hydroxyethyl)terephthalate (MHET), terephthalic acid (TPA) and ethylene glycol (EG) is far more accurate (Fig. 1A). Here, the assessment of enzyme activity using reversed-phase high performance liquid chromatography (RP-HPLC) is the most specific and prevalently used method (11–15). Furthermore, isothermal titration calorimetry-based technology was published as a sophisticated method allowing direct assessments of the enthalpy of the ester hydrolysis (16). Furthermore, Fenton chemistry-mediated fluorometric detection assay for TPA in has also been reported previously, allowing high-throughput screening of large numbers of samples (17–19).

However, these common technologies are often bound to rather high sample purities and/or high operation costs and durations. Another promising approach is therefore the use of biosensors, i.e., modified organisms, which generate a measurable readout upon encountering a specific analyte molecule.

Biosensors have proven to be highly reliable and ultrasensitive tools for the detection of a large number of molecules and are versatile tools for applications such as ultrahigh-throughput screenings (20). To date, only three functional biosensor systems for TPA have been described. Recently, Pardo and colleagues reported on the construction of a reporter strain in Acinetobacter baylyi based on superfolder green fluorescent protein (sfGFP) and the transcription factor TphR (21). In their study, Pardo et al. set out to achieve TPA conversion in A. baylyi by heterologous expression of catabolic and transporter genes from *Comamonas* sp. and identified a novel transporter capable of TPA uptake in A. baylyi. The established reporter strain was employed to evaluate the TPA uptake into A. baylyi and is able to sense concentrations as low as 10 μM.

More recently, Li and colleagues reported on the construction of a reporter strain based on the promiscuous XylS regulator protein from Pseudomonas putida (22), which is responsible for the transcription activation of the meta-toluate degradation pathway (23). In their study, Li et al. produced two variants of XylS, which could be used for TPA induced gene transcription in E. coli. When fused with sfGFP, the detection limit of the XylS-based reporter was as little as 10 μM TPA.

Furthermore, the luciferase LuxAB from Photorhabdus luminescens was employed to establish a TPA sensitive reporter system (24). The authors of this study combined a carboxylic acid reductase (CAR) and the luciferase LuxAB to generate a luminescent readout in E. coli, which semiquantitatively correlated with TPA concentration and was suitable for high-throughput screening. The reported strain was able to sense TPA concentrations as low as 1 mM.

Within this framework, we report the construction of a TPA biosensor with a higher sensitivity based on the Gram-negative bacterium *C. thiooxidans* strain S23 (DSM17888). *C. thiooxidans* is capable to take up TPA by employing the tripartite tricarboxylate transporter (TTT) family genes *tpiBA* together with the periplasmic substrate binding protein TphC and subsequent catabolism through the genes encoded in the *tph* operon (25, 26). We made use of a construct containing the TPA sensitive *tphC* promoter fused to sfGFP in order to obtain fluorescent readouts in *C. thiooxidans*. Furthermore, we prove that a deletion mutant of *C. thiooxidans* lacking the catabolic *tph* genes is a versatile reporter platform to monitor TPA concentrations as low as 1 nM. This makes it-to our knowledge-the most sensitive TPA biosensor which is reported so far.

## RESULTS

Previous work demonstrated that the Gram-negative soil bacterium *C. thiooxidans* is capable of degrading TPA using a dioxygenase and dehydrogenase for the initial conversion of TPA to protocatechuate (PCA), which is then converted further via PCA 4,5 cleavage pathway (25–29). To achieve this, the *C. thiooxidans* genome is equipped with up to two operons coding for the oxygenase proteins TphA2, A3, B, and A1 and a specific substrate binding protein, designated TphC. *C. thiooxidans* strain S23, however, encodes only for a single operon within its 5.3 Mb genome (30) (Fig. 1B). The TPA responsive regulator TphR is encoded upstream in the opposite direction of *tphC* and a promoter operator sequence of 109 bp separates both genes. Earlier work had shown that the genes *tphC-A1* are coexpressed in response to TPA and that the expression is presumably controlled by the constitutively expressed regulator protein TphR (25).

### Transcriptomic analysis confirms high TPA-dependent upregulation of genes in tph operon.

As a first step toward the construction of a TPA-sensitive reporter strain, we set out to assess other possible genes that could be upregulated in *C. thiooxidans* S23 in the presence of TPA. Therefore, we compared expression levels of genes in the presence of either TPA or succinate as a sole carbon source. Succinate was chosen due to its presence in the core metabolic pathway of the citric acid cycle and due to previous use as the substrate in minimal growth media (26). We sequenced the RNA transcripts of *C. thiooxidans* S23 grown on Wx minimal salt medium supplemented either with TPA (10 mM) or succinic acid (10 mM). Differential expression analysis of the two growth conditions showed that among the most strongly and significantly upregulated genes were those downstream of the *tphC* promoter, with *tphC* being the most upregulated with a log_2_-fold ratio of 6.46, corresponding to an 88.03-fold increased expression. Besides *tphC*, few other genes were strongly upregulated among which were also the remaining genes of the *tphC-A1* operon with *tphA1* being the least upregulated, having a log_2_-fold ratio of 3.66 corresponding to a 12.66 -fold higher expression compared to growth on succinate (Table S1, Fig. 1D).

### Cloning and assessment of the TPA reporter construct pBBR1MCS-P*_tphC_*::sfGFP (pReporTPA) in *C. thiooxidans S23*.

Based on these findings and the earlier reports on the regulation of *tphC* (25, 26), we set out to establish a TPA reporter strain based on the TPA inducible promoter of *tphC*. Therefore, the 109 bp intergenic region between *tphR* and *tphC* was amplified from genomic DNA isolated from *C. thiooxidans* S23 (Table 1) using PCR with primers RF_pCT_pGA1K::sfGFP_FW and RF_pCT_pGA1K::sfGFP_RV (Table 2). The obtained PCR product was cloned into the plasmid pG1AK_sfGFP upstream of the *sfGFP* gene. The resulting P*_tphC_*::sfGFP construct was transferred into the broad host-range plasmid pBBR1MCS-1 using the primers RF_pCT::sfGFP_pBBR1MCS_FW and RF_pCT::sfGFP_pBBR1MCS_RV. The construct pBBR1MCS-P*_tphC_*::sfGFP was verified by DNA sequence analysis for correctness and transformed into *C. thiooxidans* via electroporation. The construct was designated pReportTPA (Fig. 1C) and *C. thiooxidans* S23 carrying it was designated ReporTPA_UHH03 (Table 1). Positive transformants were identified by their resistance to chloramphenicol (Cm) and by colony-PCR using primers M13-20 fw and M13-20 rv (Table 2).

**Table 1:** Bacterial strains and plasmids used in this work

Strain	Properties	Reference/source
E. coli DH5α	*supE*44 Δ*lacU*169 (Ф80 lac*Z* ΔM15) *hsdR*17 *recA*1 *endA*1 *gyrA*96 *thi*-1 *relA*1	Invitrogen, Karlsruhe, Germany
E. coli WM3064	*thrB1004 pro thi rpsL hsdS lacZ*ΔM15 RP4–1360 Δ(*araBAD*)*567* Δ*dapA1341*::(erm pir[wt])	W. Metcalf, University of Illinois, Urbana-Champaign
E. coli *BL21* (DE3)	F-, *ompT, hsdS B (rB- m B-) gal, dcm*, λDE3	Novagen/Merck Darmstadt, Germany
*C. thiooxidans* S23	Wildtype strain capable to metabolize TPA (DSM 17888)	DSMZ, Braunschweig, Germany
*C. thiooxidans* S23_UHH01	*C. thiooxidans S23* Δ*tphA2A3BA1*, does not metabolize TPA	This work
ReporTPA_UHH03	*C. thiooxidans S23 wild-type*, carrying pReporTPA, Cm^R^	This work
ReporTPA_UHH04	*C. thiooxidans* S23_UHH01, carrying pReporTPA, Cm^R^	This work
Vector		
pG1AK-sfGFP	Modular shuttle vector for *Geobacillus* and E. coli. Used as the template for sfGFP gene	(44)
pBBR1MCS-1	Broad host-range cloning vector, *Cm^R^*, mob	(45, 46)
pReporTPA	*pBBR1MCS-1*, carrying P*_tphC_::sfGFP*	This work
pNPTS138-R6KT	Suicide plasmid backbone for allelic exchange used for gene knockout, km^R^	(47)
pNPTS138-R6KT-AB-CmR	pNPTS138-R6KT *km^R^* exchanged to *Cm^R^* and genetic regions A and B flanking TPA catabolic genes in *C. thiooxidans* strain S23.	This work
pET21a(+)::PET40	Expression plasmid for production of recombinant his-tagged PET40 PETase, Amp^R^	This work
pET21a(+)::LCC	Expression plasmid for production of recombinant his-tagged Leaf Compost Cutinase (LCC), Amp^R^	This work
pMal-p4x-*Is*PETase	Expression plasmid for production of recombinant Ideonella sakaiensis PETase fused to maltose binding protein for purification, Amp^R^	Provided by S. Weigert and B. Höcker (University of Bayreuth)

**Table 2:** Primers used in this work^*a*^

Primer	Sequence (5′ → 3′)	Length (bp)	*T_m_* (°C)
RF_pCT_pG1AK::sfGFP_FW	CAGGTCGACCTCGAGTACGCAGCGACCAAATCAAGGTGT	39	T:56 P:60
RF_pCT_pG1AK::sfGFP_RV	ACAGCTCTTCGCCTTTACGCATCTTGTCTCCTTCTTGTGTGGG	43	T:57 P:60
RF_pCT::sfGFP_pBBR1MCS_FW	CGTAATACGACTCACTATAGGGCGAATTGAGCGACCAAATCAAGGTGT	48	T:56 P:60
RF_pCT::sfGFP_pBBR1MCS_RV	ACCCTCACTAAAGGGAACAAAAGCTGTCATTTGTACAGTTCATCCATACCA	51	T:56 P:60
M13-20 fw	CCCAGTCACGACGTTGTAAAACG	23	62
M13-20 rv	AGCGGATAACAATTTCACACAGG	23	59
RF_CmR_in_pNPTS_FW	TCTGCCAGTGTTACAACCAATTAACCTTAATGAATCGGCCAACGCG	47	T:57 P:60
RF_CmR_in_pNPTS_RV	ATAAAAATATATCATCATGAACAATAAAACTGTCTTGATCGGCACGTAAGAGG	53	T:55 P:55
RF_FA_FW	CCAAGCTACGTAATACGACTCACTAGTCACACAAGAAGGAGACAAGATGC	50	T:57 P:59
RF_FA_RV	CGGCTTCAATTGCACGGGCCCCGTTAGAGCTTGACGTTGGCG	42	T:57 P:66
RF_FB_FW	GCCGCCAACGTCAAGCTCTAACTAGAAGTAGCTATCCATGCGATATC	47	T:55 P:62
RF_FB_RV	CGGCTTCAATTGCACGGGCCCCTTGGCTGGGGCCTATCAC	40	T:57 P:66
Ct_ko_seq_FW	ATGGCCCAGGCTCTGTCA	20	58
Ct_ko_seq_RV	GAGAATGGCTGACTCAGCAC	18	59

*^a^T_m_* values of restriction-free cloning primers are specified regarding the temperature used for amplification of the target fragment (T) and the temperature for insertion of the fragment into the target plasmid (P). All primers were synthesized by Eurofins MWG (Elsberg, Germany).

**FIG 2:**
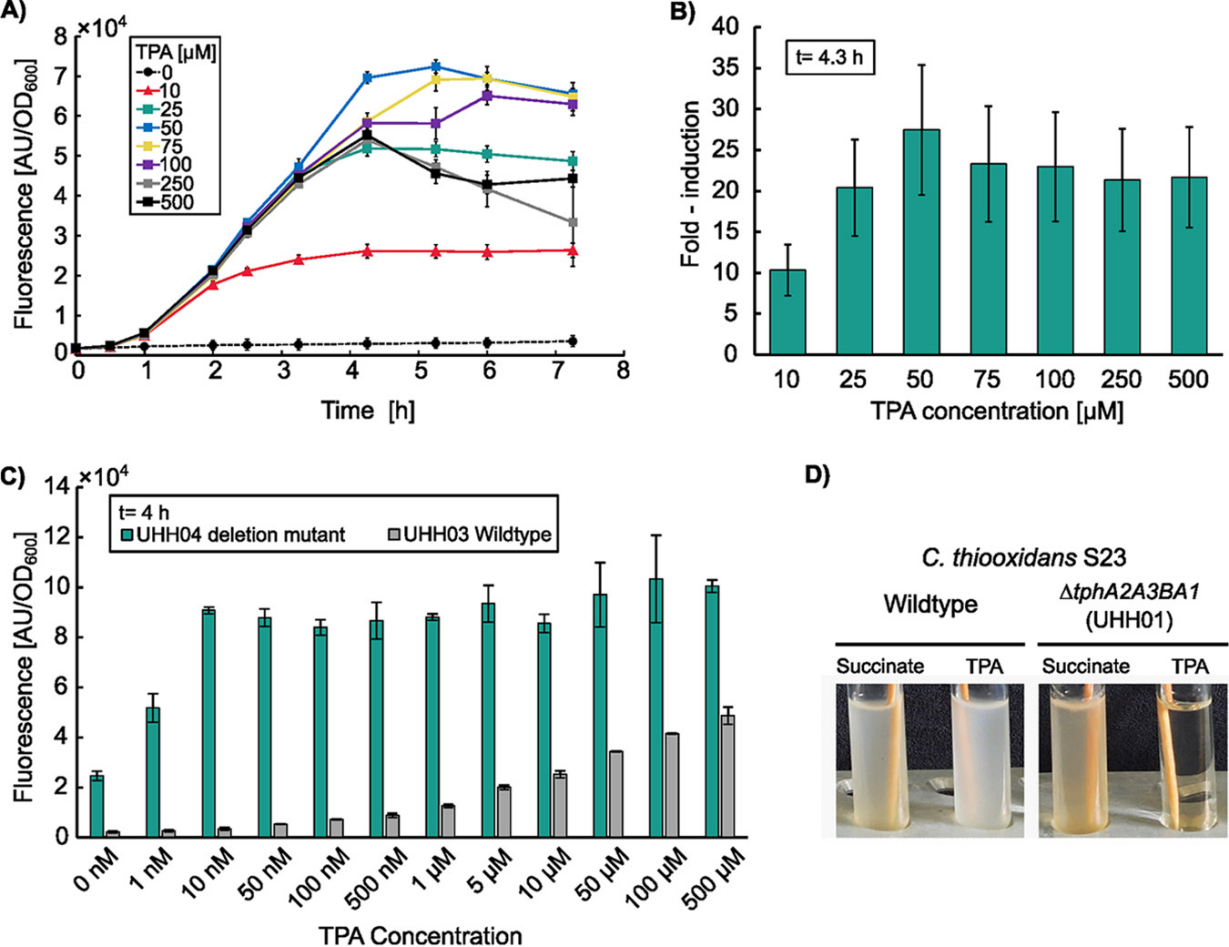
Effect of TPA on *C. thiooxidans* S23 wild type and deletion mutant carrying pReporTPA (pBBR1MCS-P*_tphC_*::sfGFP). (A) TPA-induced fluorescence response in the *C. thiooxidans* S23 parental strain harboring pBBR1MCS-P*_tphC_*::sfGFP (ReporTPA_UHH03). TPA concentrations ranging from 0 to 500 μM were added at time point 0 h and fluorescence was recorded at the different time points as described in Materials and Methods. Fluorescence signals were normalized with respect to the absorbance of the reporter cells at 600 nm. Error bars and data points represent mean values of 9 measurements. (B) Fold-induction of ReporTPA_UHH03 at the 4.3 h time point in relation to the amount of TPA added. (C) TPA-induced fluorescence response in *C. thiooxidans* S23 harboring pBBR1MCS-P*_tphC_*::sfGFP (pReporTPA) in the parental strain and the deletion mutant UHH04 (Δ*tphA2A3BA1)*. Green bars indicate the deletion mutant, gray bars indicate the wild-type strain harboring pBBR1MCS-P*_tphC_*::sfGFP (pReporTPA). Fluorescence signals were normalized with respect to the absorbance of the reporter cells at 600 nm. Error bars represent mean values of a minimum of 4 measurements. (D) Growth phenotypes of *C. thiooxidans* S23 wild type and UHH01 deletion mutant in minimal media containing either succinate or terephthalic acid (TPA) as sole carbon source. UHH01 lacks the capability to grow on TPA due to deletion of the *tphA2A3BA1* catabolic gene cluster. Cell cultures were grown in triplicate overnight at 37°C under aerobic conditions.

In order to test the responsiveness and sensitivity of ReporTPA_UHH03 toward TPA, the cells were grown in LB medium and subsequently resuspended in Wx minimal medium containing different TPA concentrations ranging from 0 to 500 μM. The fluorescence response of the cells over time was recorded (Fig. 2A).

As expected, increased TPA concentrations were positively correlated with fluorescence signals. Usually, an increase of the fluorescence signal could be observed after 1 to 2 h of aerobic incubation in 96-well black-walled microtiter plates. After 4 h of incubation, the gain in fluorescence usually flattened. Here, we observed significant differences in fluorescence signals when we used concentrations of 10 μM TPA or higher. A 10 μM TPA concentration resulted in a 10-fold increased induction based on the obtained fluorescence signal compared to the absence of TPA. A 50 μM concentration resulted in a 25-fold overall induction in ReporTPA_UHH03 (Fig. 2B). Higher concentrations did not result in a further increased fold-induction.

### Deletion of the catabolic genes *tphA2, A3, B*, and *A1* in *C. thiooxidans* S23 results in increased TPA sensitivity.

In order to further increase the sensitivity of the biosensor, a 3,677 bp deletion of the catabolic TPA genes was performed via two-step allelic exchange. The resulting deletion mutant *C. thiooxidans (*Δ*tphA2A3BA1*) was designate*d C. thiooxidans*_UHH01. As expected, *C. thiooxidans*_UHH01 lacking the *tphA2-A1* genes failed to grow on TPA (Fig. 2D). Sequencing of *C. thiooxidans*_UHH01 genomic DNA confirmed the deletion of the targeted genes (Fig. S1).

*C. thiooxidans*_UHH01 was transformed with pReportTPA via electroporation and the obtained reporter strain was designated ReporTPA_UHH04 (Table 1). Comparison of the fluorescence responses of wild-type and mutant biosensors in TPA concentrations ranging from 1 nm to 500 μM revealed a up to 10,000-fold increase in sensitivity of UHH04 compared to a parental strain carrying the same reporter construct. Even at 1 nM TPA concentration, the ReporTPA_UHH04 mutant biosensor showed a more than 2-fold increase in fluorescence compared to cells incubated without TPA. The maximum signal intensity was achieved at a 50 nM TPA concentration. Higher TPA concentrations did not result in an increased fluorescence (Fig. 2C).

**FIG 3:**
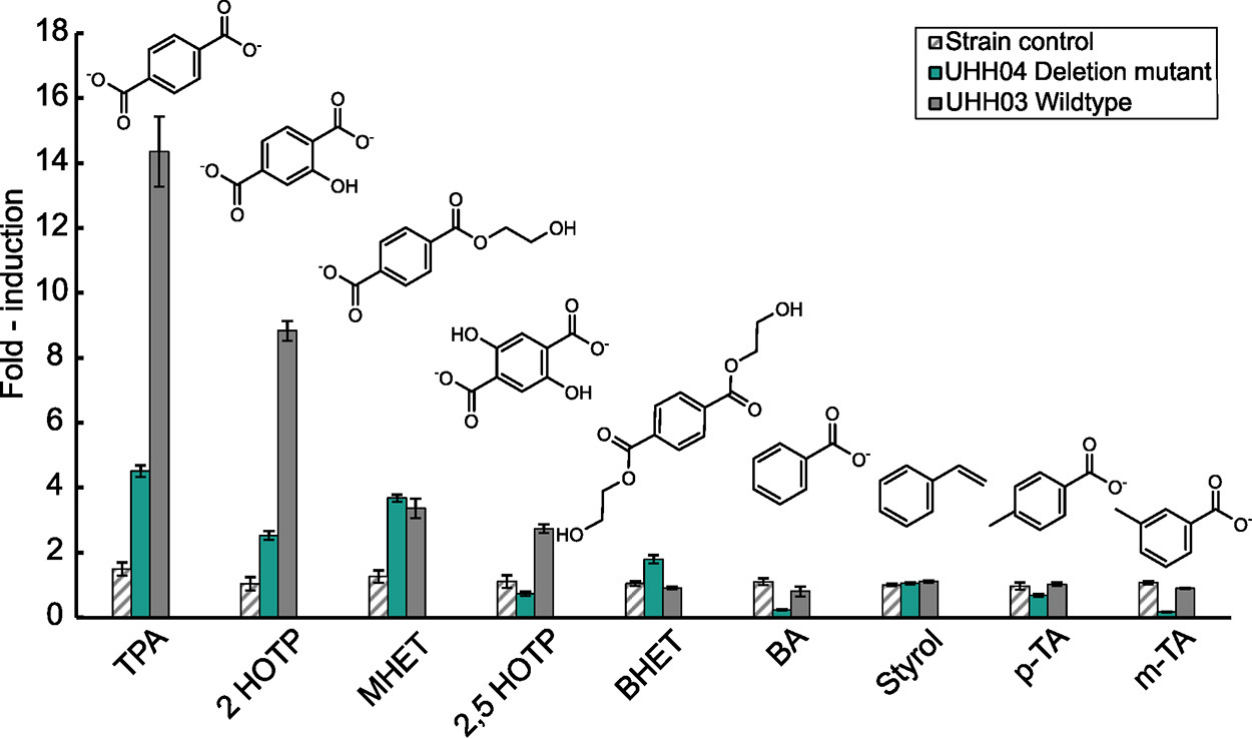
Fluorescence response of *C. thiooxidans* carrying pBBR1MCS-P*_tphC_*::sfGFP (wildtype (UHH03) and Δ*tphA2A3BA1* deletion mutant (UHH04)) toward TPA and structurally similar compounds at a concentration of 1 mM. Highest analyte specificity is shown for TPA which is the native inducer of the *tphA2A3BA1* cluster. Other compounds tested were: 2-hydroxytrephthalate (2 HOTP), mono-(2-hydroxyethyl)terephthalic acid (MHET), 2,5-dihydroxytrephthalate (2,5 HOTP), bis-(2-hydroxyethyl)terephthalate (BHET), benzoic acid (BA), styrol, p-Toluic acid (p-TA) and m-Toluic acid (m-TA). All measurements were carried out in triplicate after an incubation time of 4h. Standard deviations are given as error bars. Fold-induction values were normalized to reporter strain (wild type or Δ*tphA2A3BA1*, respectively) incubated in Wx medium without added analyte. *C. thiooxidans* Wild type carrying a pBBR1MCS-1 Backbone was employed as strain control.

We then asked how specific the regulator TphR would recognize TPA versus other possible PET degradation products and structurally similar molecules. Therefore, incubations with nine different substances in part structurally related to TPA were conducted and the fluorescence response of the biosensor strains were evaluated (Fig. 3). Interestingly, the responsiveness toward compounds which are not directly linked to PET and its degradation products rapidly declined. Notably, hydroxylated forms of TPA such as 2-hydroxytrephthalate (2 HOTP) and 2,5-dihydroxyterephthalate (2,5 HOTP) which are not directly associated with PET yielded smaller but clear responses of the reporter system.

**FIG 4:**
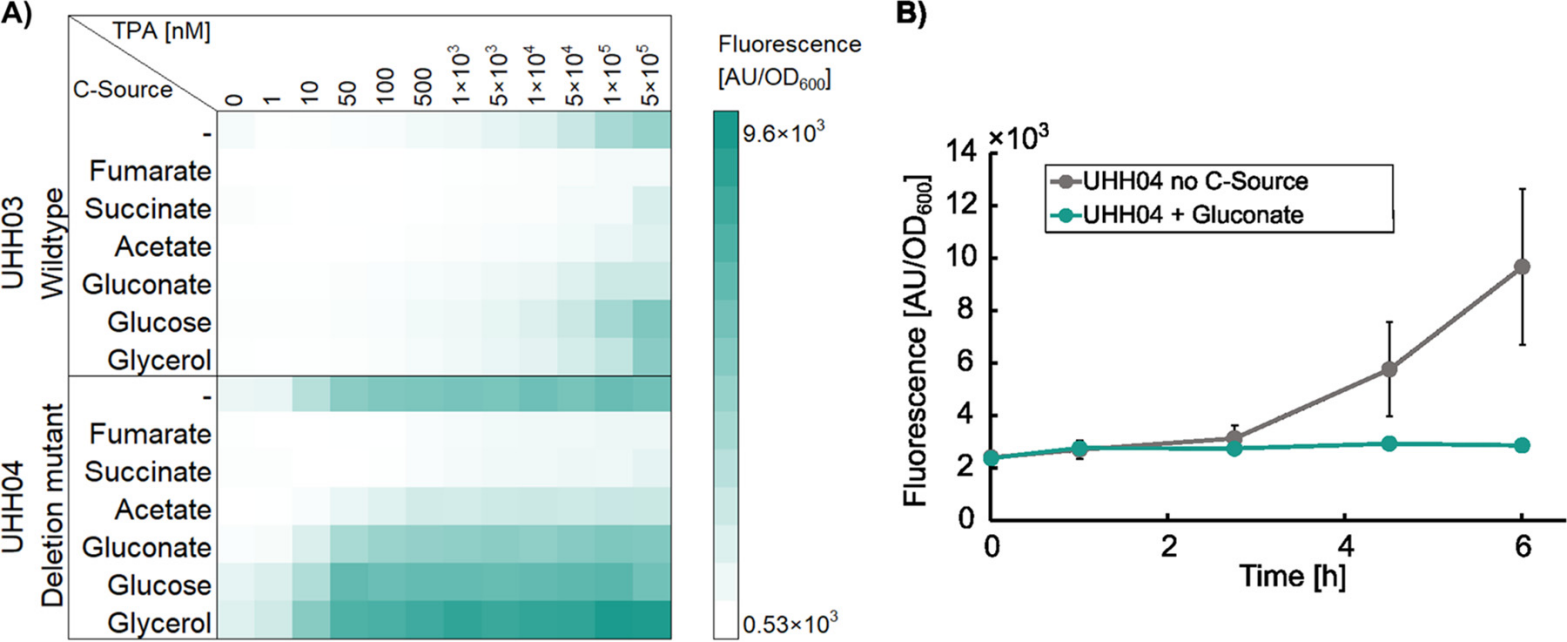
Influence of different carbon sources on the TPA induced fluorescence response in *C. thiooxidans* wild type and *ΔtphA2A3BA1* carrying pBBR1MCS-P*_tphC_*::sfGFP. (A) Heatmap showing the influence of 6 different carbon sources (5 mM) at different TPA concentrations from 0 to 500 μM. Fluorescence was recorded after 6 h of incubation. Data are mean values of three independent incubations and measurements. (B) *C. thiooxidans* (Δ*tphA2A3BA1)* carrying pReporTPA (UHH04) unspecific fluorescence increase in the absence of TPA over time. Addition of gluconate (5 mM) to the medium reduces unspecific fluorescence. Data represent mean values of three measurements per condition and time point.

Further tests with ReporTPA_UHH04 implied a slight but nonspecific increase in background fluorescence over time for cells that were grown in Wx medium in the absence of TPA. We speculated that this could be due to metabolic stress which we tried to evade by adding an alternative carbon source. Therefore, we tested the influence of 6 different carbon sources (glucose, gluconate glycerol, acetate, fumarate and succinate) on the TPA screening sensitivity and background in UHH04. The different carbon sources were supplemented at a final concentration of 5 mM to Wx medium containing 0 to 500 μM TPA. Notably, the addition of 5 mM gluconate almost completely quenched the increase in unspecific background fluorescence while maintaining a high sensitivity (Fig. 4) giving fold-induction values of up to 19 (c_TPA_ = 100 μM, *t* = 6 h).

Altogether, these data imply that the here generated *C. thiooxidans* S23 reporter strains carrying pReporTPA are specific and highly sensitive tools for the detection of PET degradation products.

### Detection of TPA by using ReporTPA_UHH04 in enzyme assays.

**FIG 5:**
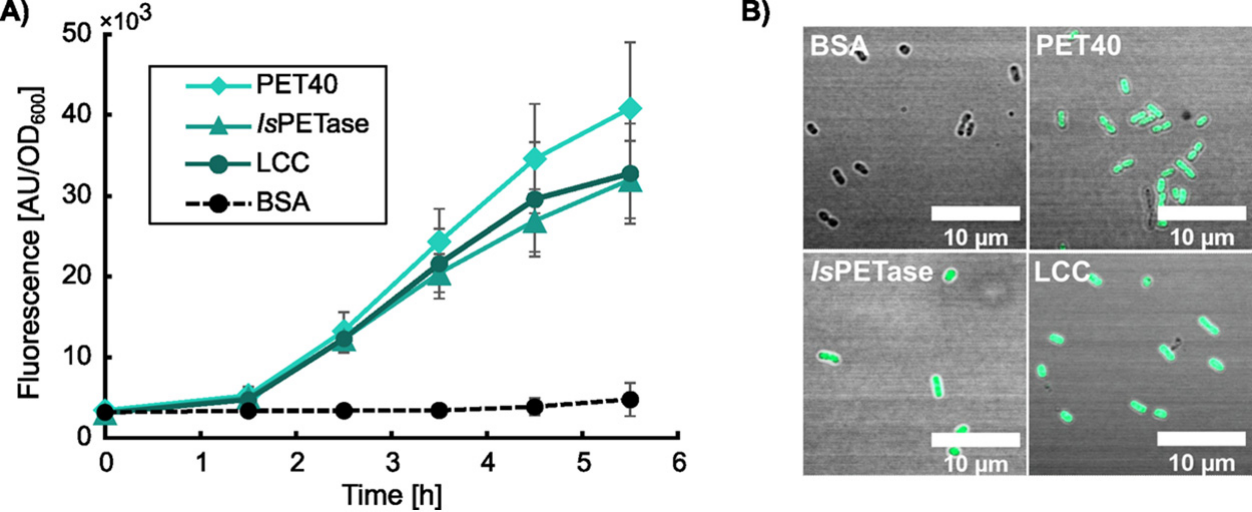
Measurement of PETase activity on PET foil substrates using deletion mutant based reporter strain ReporTPA_UHH04. (A) Fluorescence response of UHH04 on supernatants of incubations of three different enzymes with known activity on PET (*Is*PETase (11), LCC (12), PET40 (unpublished data)) on PET substrates. An incubation of BSA on PET under the same conditions served as negative control. Enzymes and BSA were incubated for 20 h at a concentration of 0.1 mg mL^−1^ in Potassium phosphate buffer (0.1 M, pH 8) prior to addition of UHH04 to reaction supernatants. (B) Fluorescent-microscopic images of UHH04 reporter cells incubated with supernatants of the enzymatic reactions of *Is*PETase, LCC, PET 40 and BSA on PET, where the incubation with BSA served as negative control. GFP channels of the pictures were obtained at and set to the same intensities to allow for comparison. Measurements and incubations were carried out in sextuplicate. Standard deviations are given as error bars.

To verify the practical usefulness of ReporTPA_UHH04, further tests were conducted in order to show the feasibility to detect PETase activity on PET substrates. Therefore, recombinantly produced and purified PETases were incubated together with PET foil as the substrate. We employed the known enzymes *Is*PETase (11) and LCC (12) together with a novel PETase (PET40), which was recently discovered by our laboratory. The reaction mixtures were incubated in a volume of 200 μL for 20 h at 40°C and after this time period, reaction supernatants were assayed using ReporTPA_UHH04 reporter cells (OD_600_ of 0.6 in Wx medium) and high pressure liquid chromatography (HPLC). The fluorescent readouts implied that TPA from PET degradation was already detectable after 1.5 to 2.5 h of incubation using ReporTPA_UHH04 (Fig. 5A). Fluorescence was furthermore detectable and distinguishable from the negative control using fluorescence microscopy (Fig. 5B) Only slight differences were observed between the different PETases. Signals obtained for the BSA control were clearly distinguishable from the PETase samples. HPLC measurements revealed TPA concentrations in the reaction supernatants of 2.74 ± 0.77 μM, 18.99 ± 2.48 μM and 6.68 ± 1.88 μM for PET40, *Is*PETase, and LCC, respectively. No TPA was detected in the Incubations of PET with BSA. Altogether, these data imply that ReporTPA_UHH04 is a very sensitive reporter strain useful for the qualitative detection of enzymatic activity on PET.

## DISCUSSION

One of the greatest challenges of our time is the environmental pollution by plastic waste. It is therefore an urgent matter to find out to what extent microorganisms and their enzymes can degrade synthetic polymers. Until now, the search for PET-degrading enzymes has mainly relied on enrichment cultures and on homology-based searches using simple sequence searches or more elaborated HMM profiles to identify possible plastic degrading enzymes (13, 31, 32). As a result of this research, a significant number of enzymes active on PET, ester-based polyurethane (PUR) and polyamide (PA) oligomers have been detected (see PAZy.eu). While the identification of these enzymes and their host microorganisms has greatly advanced our understanding in the catalytic mode of hydrolysis, we still have large knowledge gaps on the biological function, the diversity and evolution of such enzymes. With this respect, the development of functional assays to measure their activities *in situ* and to enrich the diversity of plastic-active enzymes by detecting truly novel enzymes by detecting their reaction products is an important field of research.

Within this study, we have constructed *C. thiooxidans* reporter strains ReporTPA_UHH03 and UHH04 that are highly sensitive toward the detection of TPA. They strongly transcribe a sfGFP gene fused to the *tphC* promoter with an up to 25-fold induction for UHH03 and up to 19-fold induction for UHH04 with added gluconate. The most sensitive reporter strain produced in this work, ReporTPA_UHH04, carries a deletion of the TPA degradation genes and is not able to convert TPA as a carbon source. Therefore, TPA accumulates in the cells and is responsible for the relatively high sensitivity.

Today, several methods are known to assess the degradation of PET by enzymes (14). Table 3 summarizes the most frequently used methods to assess microbial PET degradation. While some methods simply aim at measuring weight loss, they bear the risk of misinterpretation of the degradation. This is linked to the presence of additives in most polymers. More sophisticated methods aim at the direct or indirect quantitative detection of the breakdown products. The breakdown products of PET like TPA, MHET, and/or BHET are commonly analyzed by RP-HPLC analysis (11, 15). However, other approaches relying on the absorption (33) or fluorescence (17–19) of PET breakdown products have also been reported. One of the earliest fluorometric assays reported was based on iron autoxidation-mediated generation of free hydroxyl radical (18).

**Table 3:** Current methods used to assay PET degradation mainly monitoring hydrolytic TPA release

Detection method	Structural/ functional traits	Reported or assumed detection limit	Reference
Physical			
Weight loss	Mass loss of substrate due to enzymatic degradation	Not reported, mg range (>10−100 μmol TPA)	(48, 49)
Microscopy	Observation of surface changes using various microscopy techniques	Not reported/ Only qualitative assessment	See references in (6, 14)
Impedance spectroscopy	Impedance measurement over a PET membrane subjected to enzymatic degradation	μM range	(50)
Isothermal titration calorimetry	Tracking of heat generated by hydrolytic degradation of PET	~100 nM range	(16)
Chemical			
Titrimetric	Evaluation of hydrolytic activity by consumption of neutralizing agent to keep static pH throughout reaction	μM range	(51, 52)
Phenol-red pH change	Monitoring of pH change through hydrolytic depolymerization via Phenol Red dye indicator.	μM range	(53)
Photometric			
UV-absorption	UV-absorption measurement for quantification of entirety of aromatic degradation products	Lower μM range	(33)
Fenton chemistry-mediated fluorometric detection	Hydroxylation of TPA by Fenton reagent yielding fluorescent 2-hydroxyterphthalate	Lower μM range	(17–19)
Reverse phase UHPLC/HPLC	Chromatographic separation of main degradation products BHET, MHET and TPA with subsequent detection *via* UV-absorption	>1μM TPA/ MHET/ BHET	(11–13, 15)
Biological			
Halo formation in agar plates	End point analysis of overall hydrolysis of added PET nanoparticles in bacterial solid media (turbidimetric analysis)	None, not very sensitive	(13, 32, 54)
E. coli BL21 (DE3)-biosensor strain	*Photorabdus luminescens* luciferase (*luxAB)* genes combined with carboxylic acid reductase from Mycobacterium marinum to produce a chemiluminescent signal	>1 mM TPA	(24)
E. coli *BL21 (DE3)* pUC57-XylS-sfGFP	Promiscuous XylS from Pseudomonas putida was engineered to bind TPA; gene sequence of xylS and its promoter fused with sfGFP; pUC57-XylS-sfGFP	>10 μM TPA	(22)
Acinetobacter baylyi *ADP1, biosensor*	TphR (TPA responsive activator) promoter gene sequence fused with sfGFP	>10 μM TPA	(21)
ReporTPA UUH03, biosensor	pReporTPA plasmid carrying *tphC* promoter fusion with sfGFP in wild-type strain *C. thiooxidans* S23	>10 μM TPA	This work
ReporTPA UHH04, biosensor	pReporTPA plasmid carrying *tphC* promoter fusion with sfGFP in Δ*tphA2A3BA1* deletion mutant of *C. thiooxidans* S23	>1 nM TPA	This work

Furthermore, previous research has identified regulatory circuits and genes involved in the uptake of few of the polymer degradation products. Such genes and especially their promoters can be harnessed to generate very specific biosensors (Table 3). For instance, several transporters for TPA uptake had been reported (7, 21, 26) together with pathways for its degradation (7, 25) Further, a very recent study hitchhiked this principle and developed a biosensor for TPA using the transcription factor XylS from Pseudomonas putida (22). By using several rounds of mutagenesis, this transcription factor was optimized to recognize TPA at 10 μM concentration. Yet another study reported that the fusion of a *luxAB* operon for online monitoring of a chemiluminescent signal allows the detection of TPA in living cells (24). All these reporter strains build the basis for novel function-driven searches and will allow to identify novel biodiversity.

Notably, bioassays vary greatly in their sensitivity. With respect to bioassays for the detection of PET, the here developed reporter strain ReporTPA_UHH04, is probably the most sensitive bioassay for TPA reported so far (Table 3). Furthermore, the strains and constructs presented in this work can be constructed and employed relatively easy by simple 1:1 dilution with liquid analytes such as enzymatic reaction supernatants providing fluorescent readouts already after 2 to 4 h of incubation. This offers a great potential to identify novel PET-active microorganisms and enzymes even with low activities. It also offers the possibility to monitor the degradation in microbial consortia over time and *in situ*. Due to the accumulation of TPA over time in the ReporTPA_UHH04 cells, we could non quantitatively detect the release of TPA from PET by different PETase enzymes. Assays were performed already after a relatively short incubation time of only 20 h on PET, releasing lower micromolar amounts of TPA. Additional developments could be focused on modification of screening conditions to further shorten the assaying durations and possibly enabling quantitative evaluation of PET breakdown.

Future work will now have to demonstrate the usefulness of the ReporTPA system for environmental and biotechnological studies. Thereby, identifying novel and better PET degrading microorganisms and enzymes will be a rewarding task. While bioassays are now available for the detection of PET breakdown products, we lack such assays for polymers like polyethylene, polypropylene, polystyrene, polyvinyl chloride and ether-based PUR. Thus, developing bioassays to monitor the possible degradation of these polymers is one of the next major challenges.

## MATERIALS AND METHODS

### Culturing conditions.

Bacterial strains and plasmids used in this study are listed in Table 1. E. coli strains were routinely cultivated in lysogeny broth (LB) medium (34) supplemented with the appropriate antibiotics at 37°C. *Comamonas thiooxidans* strains were cultivated either in LB or in Wx mineral medium (35) at 37°C, if not stated otherwise.

### Molecular cloning.

Synthesis of the gene coding for PET40 (IMG Gene ID: Ga0074072_10050165) was done by Biomatik (ON, Canada). Cloning of genetic fragments from different sources into target plasmids was routinely performed through restriction-free cloning (36) using the corresponding primers listed in Table 2. Genetic constructs were either transformed into chemically competent E. coli DH5α cells through heat shock at 42°C for 45 s or via electroporation into electrocompetent *C. thiooxidans* cells (1350 V, 600 Ω, 10 μF, 1 mm). Electrocompetent cells were prepared by growing a 10 mL LB culture until an OD_600_ of 0.3 was reached followed by aliquoting the cells into 1 mL and washing them by resuspending the centrifuged cells three times with 1 mL ice-cold 10% (vol/vol) glycerol. Cells were finally resuspended in 100 μL 10% (vol/vol) glycerol and stored at −70°C until use.

### Plasmid and genomic DNA isolation.

Plasmid DNA for use as the template DNA in PCRs or for transformation was routinely isolated from E. coli DH5α cells using the Presto Mini Plasmid kit (GeneAid, New Taipei City, Taiwan). Isolation of genomic DNA from *C. thiooxidans* for use as the template DNA in PCRs was performed using the NucleoSpin Microbial DNA kit (Macherey-Nagel, Düren, Germany).

### Preparation of reporter cells for TPA assays.

The *C. thiooxidans* S23 biosensor strains ReporTPA_UHH03 and UHH04 were prepared by growing them overnight at 130 rpm in 50 mL LB medium supplemented with 25 μg mL^−1^ chloramphenicol in an Erlenmeyer flask.

Prior to TPA assays, the OD_600_ of the cultures was determined and a suitable culture volume was centrifuged at 4,500 rcf, 4°C for 5 min followed by resuspension of the pellet in 50 mL Wx medium containing 25 μg mL^−1^ chloramphenicol to reach a final OD_600_ of 0.6. Additionally, for experiments with ReporTPA_UHH04, the Wx medium for resuspension was supplemented with 10 mM gluconate, if not stated otherwise. The resuspended cultures were incubated at 37°C, 130 rpm for 30 min before addition to the samples.

### Fluorescence assays with *C. thiooxidans* S23 ReporTPA_UHH03 and UHH04.

For routine assaying of liquid samples comprising either Wx medium containing different TPA concentrations or supernatants from enzymatic reactions, 100 μL of sample was added to a well in a black-walled 96-well microtiter-plate for fluorescence-based assays (ThermoFisher, Waltham, MA, USA). A volume of 100 μL of reporter cells, which were prepared as described in the previous section, were added to the sample wells and the plate was incubated at 28°C on a Vibration Shaker 3023 plate shaker (Gesellschaft für Labortechnik mbH, Burgwedel, Germany) at 150 rpm. Fluorescence and OD_600_ values throughout the incubation times were determined in 0.5 to 2 h intervals using a Synergy HT plate reader using the Gen5 software (BioTek, Winooski, VT, USA).

### Fluorescent microscopic imaging of *C. thiooxidans* biosensor cells.

Microscopic imaging of biosensor cells was conducted using an Axio Observer.Z1/7 LSM 800 (Carl Zeiss Microscopy GmbH, Jena, Germany) confocal laser scanning microscope with a Plan-Apochromat 100×/1.40 Oil DIC M27 objective. Evaluation and processing of obtained images was performed using ZEN software (Version 2.3, Carl Zeiss Microscopy GmbH) and Fiji software v.1.53c (37).

### Transcriptomic and differential expression analysis of *C. thiooxidans* S23 cells.

Cultures of *C. thiooxidans* S23 wild-type cells were grown in triplicate in 250 mL Wx minimal salt medium containing 10 mM either TPA or succinate. Cells were harvested after reaching an OD_600_ of 0.5, shock frozen on dry ice and stored at −80°C until further processing. Cell pellets were sent for RNA-sequencing to Vertis Biotechnologie AG (Freising, Germany). Obtained raw reads were further processed in Geneious Prime 2021.1.1 (www.geneious.com) by quality control and trimming using BBDuk v.38.84 (38), mapping onto *C. thiooxidans* S23 (DSM17888) genome (PATRIC Genome ID: 363952.7) with Bowtie2 (39) and subsequent calculation of expression levels using Geneious Prime. Differential expression analysis was performed using DeSEQ2 v.1.36 (40). Raw reads were deposited at the ENA under project accession number: PRJEB55783.

### Two-step allelic exchange for deletion of the catabolic *tphA2A3BA1* gene cluster in *C. thiooxidans* S23.

Deletion of the catabolic *tphA2A3BA1* gene cluster in *C. thiooxidans* S23 was performed according to reference (41). Homologous regions, designated A and B, flanking the *tphA2A3BA1* genes 989 bp upstream of *tphA2 (A)* and 993 bp downstream of *tphA1 (B)* were amplified from *C. thiooxidans* gDNA using primer pairs RF_FA_FW, RF_FA_RV and RF_FB_FW, RF_FB_RV. The correct product lengths were confirmed via gel electrophoresis and the regions were joined via overlap extension PCR. The resulting fragment AB was cloned into the suicide vector pNPTS138-R6KT::CmR via whole plasmid amplification using overlaps complementary to the target vector. Due to a natural kanamycin resistance of *C. thiooxidans*, the template vector pNPTS138-R6KT::CmR was constructed by replacing the kanamycin resistance gene in pNPTS138-R6KT with a chloramphenicol resistance gene from pBBR1MCS-1 using restriction-free cloning with the primers RF_CmR_in_pNPTS_FW and RF_CmR_in_pNPTS_RV. The resulting construct pNPTS138-R6KT::CtAB::CmR was transformed into chemically competent E. coli WM3064 cells and plated on LB-agar containing 25 μg mL^−1^ chloramphenicol and 30 μM diaminopimelic acid (DAP). Positive transformants were selected via antibiotic resistance and sequencing using M13-20 fw and M13-20 rv primers. Integration of pNPTS138-R6KT::CtAB::CmR into the *C. thiooxidans* S23 genome via allelic exchange was performed via conjugation by cocultivation of *C. thiooxidans* S23 and E. coli WM3064 harboring pNPTS138-R6KT::CtAB::CmR on LB-agar. After overnight incubation at 37°C, grown cells were washed off with 2 mL LB medium, pelleted, washed two times with 1 mL LB medium and plated out on LB agar containing 25 μg mL^−1^ chloramphenicol to select for *C. thiooxidans* with genomically integrated pNPTS138-R6KT::CtAB::CmR. A second selection step for cells having lost the integrated plasmid sequence was carried out on LB-agar containing 10% sucrose for counterselection. Resulting colonies were tested for their sensitivity toward chloramphenicol and growth capability on TPA. The genomic DNA of cells unable to grow in the presence of Cm and TPA as sole carbon source was investigated by sequencing the region amplified using the primers Ct_ko_seq_FW and Ct_ko_seq_RV to verify the successful deletion of the *tphA2A3BA1* gene cluster.

### Heterologous expression of PETase genes in Escherichia coli BL21(DE3).

Recombinant protein expression for the production of PET-degrading enzymes was performed using E. coli BL21(DE3) harboring a novel PETase from our laboratory, PET40 (unpublished data) or leaf compost cutinase (LCC) (12) on pET21a(+) expression plasmids or *I. sakaiensis* PETase (11) on a pMAL-p4x expression plasmid (Table 1). Cells were grown at 37°C in 1 L ZYM-5052 auto induction medium (42) supplemented with 100 μg/mL ampicillin under constant aeration. The cultures were relocated from 37°C to 22°C once an OD_600_ of 0.6 was reached and incubated further overnight for 16 to 20 h. Cells were harvested after overnight incubation by centrifugation and lysed using a French pressure cell press. Crude lysates were cleared by centrifugation at 20,000 rcf for 20 min and the obtained supernatants were purified using either Ni-NTA agarose (Macherey-Nagel, Düren, Germany) for polyhistidin-tagged proteins PET40 and LCC or amylose resin (New England Biolabs, Ipswich, MA, USA) for *Is*PETase.

### Incubation of PETase enzymes with PET substrates.

Purified PETase enzymes were diluted to a final concentration of 0.1 mg mL^−1^ in Potassium phosphate buffer (0.1 M, pH 8) and 200 μL of enzyme solution were added to a well of a 96-Well Plate. Added PET substrates comprised of a PET foil platelet (Ø 6 mm, amorphous PET film, Goodfellow GmbH, Bad Nauheim, Germany) which was rinsed in 96% Ethanol and dried prior to addition to the well. Incubation was carried out for 20 h at 40°C on a thermomixer Comfort 5355 (Eppendorf SE, Hamburg, Germany) at 400 rpm. A 0.1 mg mL^−1^ Solution of Bovine serum albumin (BSA) in Potassium phosphate buffer (0.1 M, pH 8) served as negative control for incubation.

### HPLC analysis of enzymatic incubations with PET.

The analysis of TPA concentrations in supernatants of enzymatic incubations with PET was performed using an UltiMate 3000 UHPLC system from Thermo Scientific (Waltham, MA, USA). The system was fitted with a Triart C18 column (YMC Europe GmbH, Dinslaken, Germany) having a dimension of 100 × 2.0 mm with 1.9 μm particle diameter. Isocratic elution was carried out in a 20:80 (vol/vol) acetonitrile and water (acidified with 0.1% vol/vol trifluoroacetic acid) mobile phase at 0.4 mL min^−1^. Samples were prepared from 50 μL of incubation supernatant mixed with 200 μL acetonitrile (acidified with 1% vol. trifluoroacetic acid), followed by centrifugation at 10,000 × *g* for 3 min. 200 μL of the supernatant was transferred to 600 μL water. 15 μL of sample were injected per measurement. Detection TPA was carried out at 254 nm with a VWD-3400 detector from Thermo Scientific (Waltham, MA, USA). The quantification of peaks was performed with the data analysis software supplied with the Compass HyStar software package from Bruker (Billerica, MA, USA).

### Data availability.

Raw reads of the transcriptomic analysis were deposited at the European nucleotide archive ENA under project accession number PRJEB55783.
