# Supplementary material for: An Ultra-Sensitive Comamonas thiooxidans Biosensor for the Rapid Detection of Enzymatic Polyethylene Terephthalate (PET) Degradation
Source: Appl Environ Microbiol. 2022 Dec 12;89(1):e01603-22. doi: 10.1128/aem.01603-22 (PMC9888244; doi:10.1128/aem.01603-22)
Supplement: Supplemental file 1 — Fig. S1. Download aem.01603-22-s0001.pdf, PDF file, 0.1 MB [file aem.01603-22-s0001.pdf]

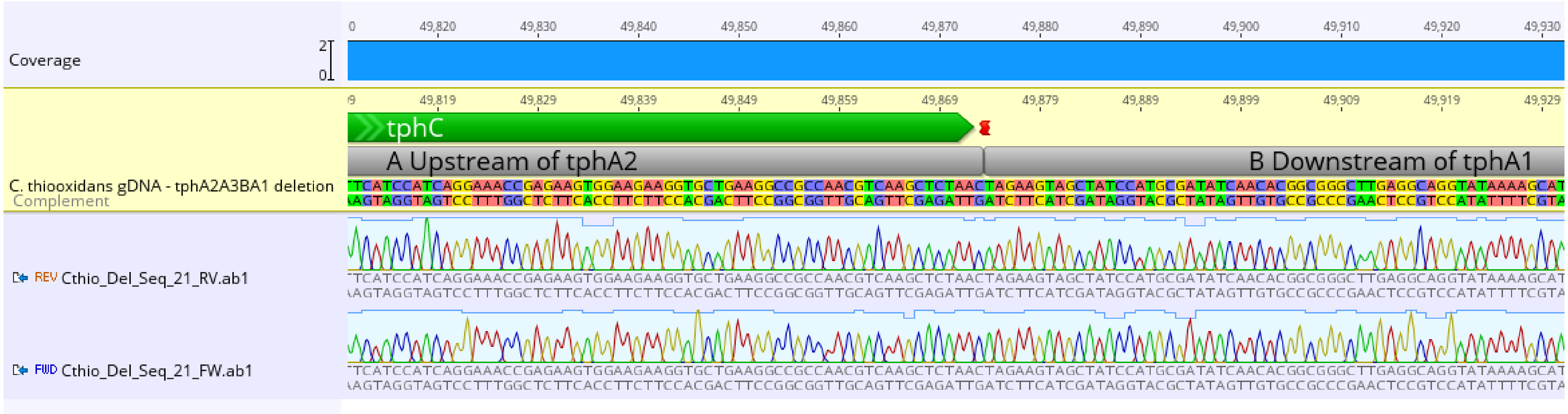

Figure S1: Sequencing of successful deletion of *tphA2A3BA1* gene cluster in *C. thiooxidans* UHH1 using primers Ct_ko_seq_FW and Ct_ko_seq_RV (TABLE 2). Figure was generated using Geneious Prime 2021.1.1 (www.geneious.com).
